# Knowledge towards Cervical and Breast Cancers among Industrial Workers: Results from a Multisite Study in Northern Vietnam

**DOI:** 10.3390/ijerph16214301

**Published:** 2019-11-05

**Authors:** Bach Xuan Tran, Tracy Vo, Anh Kim Dang, Quang Nhat Nguyen, Cuong Tat Nguyen, Chi Linh Hoang, Khanh Nam Do, Carl A. Latkin, Cyrus S. H. Ho, Roger C. M. Ho

**Affiliations:** 1 Institute for Preventive Medicine and Public Health, Hanoi Medical University, Hanoi 100000, Vietnam; quang.n.nguyen@alumni.duke.edu (Q.N.N.); donamkhanh@hmu.edu.vn (K.N.D.); 2 Bloomberg School of Public Health, Johns Hopkins University, Baltimore, MD 21205, USA; carl.latkin@jhu.edu; 3 Department of Sociomedical Sciences, Mailman School of Public Health, Columbia University, New York, NY 10027, USA; tracy.vo@columbia.edu; 4 Institute for Global Health Innovations, Duy Tan University, Da Nang 550000, Vietnam; kimanh.ighi@gmail.com (A.K.D.); cuong.ighi@gmail.com (C.T.N.); 5 UnivLyon, Université Claude Bernard Lyon 1, 69100 Villeurbanne, France; 6 Center of Excellence in Behavioral Medicine, Nguyen Tat Thanh University, Ho Chi Minh City 700000, Vietnam; chi.coentt@gmail.com (C.L.H.); pcmrhcm@nus.edu.sg (R.C.M.H.); 7 Department of Psychological Medicine, National University Hospital, Singapore 119074, Singapore; cyrushosh@gmail.com; 8 Department of Psychological Medicine, Yong Loo Lin School of Medicine, National University of Singapore, Singapore 119228, Singapore; 9 Institute for Health Innovation and Technology (iHealthtech), National University of Singapore, Singapore 119077, Singapore

**Keywords:** industrial worker, factory worker, cervical cancer, breast cancer, Vietnam, sexual health, women’s health

## Abstract

Breast and cervical cancer cases are rising among service and industrial women workers in Vietnam. We conducted a cross-sectional study among 287 workers in three factories in Hanoi and Bac Ninh from July to September 2018 to describe the knowledge of these cancers among industrial workers in Northern Vietnam using a structured questionnaire. Factors associated with knowledge of breast and cervical cancer were identified using generalized linear models (GLM). In our study, approximately one-third of participants believed breast cancer was caused by the lack of breastfeeding, exposure to pollution, and chemicals. Less than 50% knew about sexually transmitted infections that can cause cervical cancer or were aware of a vaccine for cervical cancer. Having one sexual partner within the last year was positively associated with having a higher score of knowledge for both diseases. Receiving a medical checkup within the last 12 months and seeking health information via the internet were related to greater breast cancer knowledge. Targeted education campaigns are needed to ensure proper knowledge and improve awareness of breast cancer and cervical cancer among industrial workers.

## 1. Introduction

The World Health Organization estimated that breast cancer impacts 2.1 million women each year [1], accounting for nearly a quarter (23%) of all cancers in women [2]. Moreover, although breast cancer detection technology has advanced rapidly, the mortality rate for breast cancer is still high—about 627,000 women died from breast cancer in 2018, representing approximately 15% of all cancer deaths among women [1]. Furthermore, while breast cancer rates among women in developed regions are higher, the number of breast cancer incidences has increased in both developed and developing countries [3]. We have also observed a great difference in the survival rates of breast cancer worldwide: the five-year survival rate is 80% in developed countries, but it remains under 40% in developing countries [4]. Along with breast cancer, cervical cancer is a main, life-threatening concern of public health. Cervical cancer ranks fourth among the most frequently diagnosed cancers in women [5]. In 2018, there were 570,000 new cases, which represented 6.6% of all cancers in women [5]. Notably, approximately 90% of deaths related to cervical cancer were reported in low- and middle-income countries [6]. The constraints of resources and infrastructure pose major challenges for developing countries to manage and improve both breast cancer and cervical cancer prevention and outcomes.

In Vietnam, the rate of breast cancer has dramatically risen over the previous two decades. According to the result of Global Burden of Disease study, in 2000, the incidence rate of breast cancer was 8.57 per 100,000 women, up to 12.18 per 100,000 women in 2010, and 15.7 per 100,000 women in 2017 [7,8]. This makes breast cancer the most frequently diagnosed cancer among Vietnamese women [9]. The age-standardized cervical cancer incidence rate was 8.26 per 100,000 women in Vietnam in 2000, 9.17 per 100,000 women in 2010, and up to 9.75 per 100,000 women in 2012 [8,10]. Often, according to reports from other developing countries, women lacked awareness of the causes, risks, and prevention of cervical cancer [11,12]. Moreover, the majority of patients with breast cancer and cervical cancer are detected too late, and therefore, are often diagnosed at an advanced stage of the disease, partly due to their inadequate understanding of the symptoms [13,14]. In order to reduce morbidity and mortality and to promote early screening and detection, there need to be comprehensive assessments of knowledge and awareness of breast cancer and cervical cancer.

Vietnam has had remarkable economic transformations in the past two decades [15]. Vietnam’s workforce, together with an influx of migrant and immigrant workers, has increased exponentially, especially in areas with industrial factories [16]. Previous studies showed that breast cancer and cervical cancer incidences are disproportionate among industrial workers or factory workers. For example, studies conducted in electronics factories in Taiwan and Malaysia found that women who were exposed to certain mixtures of solvents had a much greater risk of breast cancer and that breast cancer screening practices among these female workers were lower than the national average, respectively [17,18]. In terms of cervical cancer, service and apparel manufacturing workers and different services and workers in industries (such as maids, cleaners, and cooks) had an elevated risk of cervical cancer [19,20]. However, data on breast cancer and cervical cancer among factory workers in Vietnam remain limited. Our study aimed to examine the knowledge regarding causes, signs, and prevention methods of breast cancer and cervical cancer among factory workers in Vietnam. We also investigated factors associated with having knowledge about these two cancers, which together may help tailor interventions for our target population in factories.

## 2. Materials and Methods

### 2.1. Study Design

This cross-sectional study was carried out in three factories in Hanoi and Bac Ninh from July to September 2018. Hanoi and Bac Ninh are the two technology development hotspots within the largest industrial zones in Northern Vietnam. We selected a total of 289 participants by convenience sampling according to the following eligibility criteria: (1) at least 18 years old; (2) have current labor contracts with the three aforementioned factories, and (3) have worked at those factories for at least six months. Participants had to have the capacity to communicate with the interviewers. We excluded participants who suffered from severe illness during the recruitment process.

Briefly, participants who met the eligibility criteria were asked to be interviewed in the counseling room by a research member. Once participants agreed to participate in the study, they were fully informed about the purpose of the study, benefits, and drawbacks. Participants also signed written informed consents acknowledging their rights and protection of their confidentiality.

### 2.2. Measure and Instruments

A 20-minute face-to-face interview was conducted. The data collectors were program staff and researchers who underwent training. Factory staff were not involved in participant recruitment. Before the interview process, a pilot survey was carried out among 20 respondents. To examine the feasibility of recruitment, we selected workers of different ages and genders. Minor modifications in the wording of the standardized questionnaire (Supplementary Materials S1) were incorporated to meet participants’ cultural backgrounds. A structured questionnaire was developed with the following information:

#### 2.2.1. Socioeconomic Characteristics

Respondents self-reported their gender, age, educational level, marital status, immigration status, monthly income, and the number of children. We also asked participants to self-report the number of years of experience in their respective fields and the number of working hours per day.

#### 2.2.2. Knowledge of Breast Cancer and Cervical Cancer

In this study, a series of questions about knowledge about breast cancer and cervical cancer were asked regarding their causes, important signs and symptoms, and prevention methods. We developed a scoring system for the participants’ knowledge about these cancers. The scale Cronbach’s alpha value in both breast cancer knowledge and cervical cancer knowledge were 0.92. The total score of knowledge of breast cancer ranged from 0 to 18 points and knowledge of cervical cancer was from 0 to 19 points.

#### 2.2.3. Health Risk Behaviors

Regarding drinking practices, we interviewed participants based on the Alcohol Use Disorders Identification Test—Consumption (AUDIT-C) [21]. AUDIT-C consists of three questions—the lowest score was 0 and the highest score was 12. A higher score suggests a higher level of alcohol dependence. Hazardous drinking was defined as a total score of 4 or greater for men and 3 or greater for women. Binge drinkers were identified if they reported any positive responses (i.e., “often”, “very often”, “all the time”) to the question: “How often do you have six or more drinks on one occasion?”

Participants’ smoking patterns were identified based on 3 major groups: current smokers, former smokers, and never smokers. Current smoker was defined as an adult who had smoked at least 100 cigarettes during his/her lifetime and had smoked within the previous 30 days. Former smoker was defined as an adult who had smoked 100 cigarettes in his/her lifetime but without smoking in the last 30 days. An adult was classified as never a smoker if he/she had never smoked around 100 cigarettes, or had never smoked, in their lifetime.

#### 2.2.4. Health Seeking Behaviors

We collected information on the frequency of receiving medical check-ups and using reproductive healthcare services within the last 12 months, which are common methods of accessing health-related information. Participants’ number of sexual partners in the last 12 months was also collected.

### 2.3. Statistical Analysis

Researchers analyzed the data using STATA 12.0 (Stata Corp. LP, College Station, TX, USA). Socioeconomic characteristics, knowledge of breast cancer and cervical cancer, health risk behaviors, and health-seeking behaviors were presented. These variables were considered as potential covariates in the multivariate regression models. Factors associated with knowledge of breast cancer and cervical cancer were identified using generalized linear models (GLM). A *p*-value of less than 0.05 was considered statistically significant. For detecting multicollinearity, we used vif values. In our study, all vif values were less than 10, which indicated no multicollinearity between the variables.

### 2.4. Ethics Approval

This study protocol was approved by the Institutional Review Board of Hanoi Medical University (code: 01a-QD/VNCTN).

## 3. Results

Table 1 illustrates the demographics and working characteristics of the participants. The majority of respondents were female (83.3%). The majority of participants had attained high school education or above (61.3% and 30%, respectively), 95.9% of respondents were living with their spouses or partners. The mean age of respondents was 31.9 (SD = 4.5), and the mean monthly income was $273.60 (SD = 96.5). The mean number of years of experience was 9.9 (SD = 3.8), and the mean number of working hours per day was 8.2 (SD = 0.9).

Table 2 reveals that approximately two-thirds of participants had previously had sex in the last 12 months (65.9%). In addition to a difference in the current smoking status, the percentages of male participants who were identified as hazardous drinkers and binge drinkers were all greater than those of female participants. The majority of participants received medical check-ups in the last 12 months (94.1%), and the number of female workers using reproductive healthcare and premarital services in the last 12 months (48.9%) was greater than the number of males using such services (31.1%). Participants often found health information via medical staff (50.0%), followed by the internet (49.3%) and social media (47.9%).

Table 3 illustrates the participants’ knowledge regarding breast cancer. Approximately one-third of participants believed that breast cancer is caused by exposure to pollution and chemicals (39.4%), mammary gland enlargement (32.6%), lack of breastfeeding (32.3%), and smoking (29%). Moreover, the majority of participants believed the most common signs of breast cancer was feeling lumps in the breast (76.3%), followed by breast enlargement or changes in breast shape (45.9%) and swollen lymph nodes in armpits (36.9%). The majority of participants agreed that early diagnosis of breast cancer was a measure of prevention (81.3%), and about half of the participants mentioned other ways, including breastfeeding (45%), having a healthy diet (41%), and regular exercise (45.7%). The mean breast cancer knowledge score was 6.9 (SD = 5.1).

Table 4 shows the participants’ knowledge of cervical cancer. Almost 50% of participants believed that respective sexually transmitted infections and diseases caused cervical cancer: syphilis (44.7%), gonorrhea (44.0%), genital warts (41.8%), and chlamydia (41.8%). More than three-fourths of respondents agreed that genital infections result in cervical cancer (77.5%) and half of the participants believed having a lot of sexual partners was a cause of cervical cancer (50%). Over half of the participants identified vaginal bleeding after sex (54.5%) as a sign of cervical cancer, followed by having vaginal discharge (43.0%), and pain when having sex (40.9%). The majority of participants mentioned periodic gynecological examination and gynecological treatment as a measure of prevention of cervical cancer (77.6%). About half of the participants (46.6%) mentioned vaccination or not having many sexual partners as a potential preventive measure against cervical cancer.

Table 5 illustrates the factors associated with knowledge of breast cancer and cervical cancer. Female participants were more likely to have knowledge about both breast cancer and cervical cancer (Coef = 1.20; 95% CI: 0.58, 1.82; Coef = 0.94; 95% CI: 0.32, 1.56, respectively). Participants who reported having received medical checkups in the last 12 months were more likely to have higher breast cancer knowledge, compared to those who reported not having received checkups in the last 12 months (Coef = 1.73; 95% CI: 0.23, 3.24). In terms of the source where the participants obtained their health information, participants who reported receiving health information via the internet were more likely to have knowledge of breast cancer (Coef = 1.66; 95% CI: 1.09, 2.22), and those who received knowledge via friends/relatives had greater knowledge of cervical cancer (Coef = 0.30; 95% CI: 0.06, 0.55).

**Figure A1:**
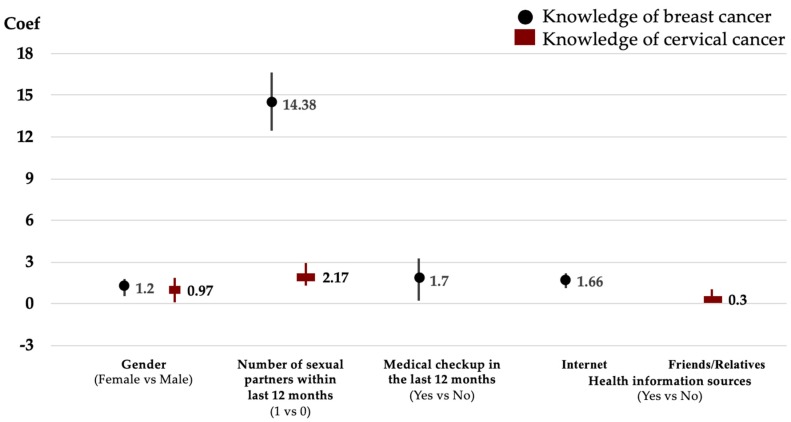
Significant factors associated with knowledge of breast cancer and cervical cancer.

Figure A1 also presents significant factors associated with a score of knowledge of breast cancer and cervical cancer.

## 4. Discussion

Our study sheds light on the perceived knowledge of breast cancer and cervical cancer, along with their associated factors, among female factory workers in Northern Vietnam. Findings from this study highlighted the low to moderate level of adequate knowledge of breast cancer and cervical cancer among the workers, especially regarding causes and common signs/symptoms of the diseases. Importantly, only half of the respondents were aware of a vaccine that could help prevent cervical cancer. In this study, we observed that medical staff and the internet were the most common sources of health information for participants. We also identified multiple factors associated with having a higher score of knowledge for breast cancer and cervical cancer. Our results provide insights for physicians and policymakers to implement targeted interventions to improve awareness about breast cancer and cervical cancer, especially among factory workers.

According to the theory of health behavior change, people will be more likely to engage in changing health behaviors by fostering their knowledge and beliefs to enhance their self-regulation skills and abilities [22]. As knowledge influences behavior-specific self-efficacy, enhancing participants’ knowledge regarding causes and symptoms may play a vital role in the participants’ ability to engage in screening and early detection of breast cancer and cervical cancer [23,24]. The majority of participants were not fully aware of the causes of breast cancer. This result is consistent with a previous study conducted in Malaysia, which revealed a low level of adequate knowledge about breast cancer risk factors [25]. Of note, although a high percentage of participants mentioned pollution and chemicals as risk factors for breast cancer, these factors are considered general risk factors for all types of cancers, and are not specific to breast cancer [26,27]. Moreover, in-depth knowledge about the signs of breast cancer was low among workers. Although about three-fourths of participants knew that feeling lumps in the breast and breast enlargement or a change in breast shape were signs of breast cancer, the proportion of participants recognizing other signs or symptoms for breast cancer was low (overall, less than 40%). Findings from other studies, especially from other low–middle income countries, showed similar results [13,28,29,30]. Compared to results from developed countries, the percentage of women in those studies who were aware of breast cancer symptoms was higher, to a certain extent, which can be explained by their greater consciousness of health and higher living standards [29,31]. As the majority of breast cancer cases are detected by patients themselves [32], it is crucial that women are fully aware of symptoms and breast self-examination techniques for early detection. In addition, knowing about causes and risk factors are essential to reducing one’s risk and actively preventing breast cancer.

This study showed that the internet was considered as the most common source of health information (ranked after medical staff) for breast cancer [33]. However, low knowledge of early breast cancer screening and detection was noted in a previous study [7]. Notably, cases of late-stage breast cancer often resulted from delayed diagnosis and a lack of awareness of the signs and symptoms [34,35]. Previous studies in fact have highlighted the important role of media-led health education interventions that promote screening for breast cancers [25,36,37]. Therefore, the internet could be exploited as a potential means for disseminating proper information about breast cancer [38]. This strategy could complement the knowledge gained from medical checkups given that participants who reported receiving regular medical checkups were more likely to have greater breast cancer knowledge.

In this study, a high percentage (77.5%) of participants knew that genital infections were a major cause of cervical cancer. However, less than half of them mentioned certain sexually transmitted infections (syphilis, gonorrhea, warts, chlamydia) and having multiple sexual partners as risk factors for cervical cancer. The limited cervical cancer knowledge may limit participants’ abilities to change lifestyle-related risk factors, as well as to seek prevention methods for cervical cancer [39]. About half of the respondents mentioned vaginal bleeding after sex as a symptom of cervical cancer, but a low proportion knew about other signs or symptoms. This figure is comparatively high compared to the results of previous studies in Ethiopia [40] and the Democratic Republic of Congo [41], but lower than those from a study in Cambodia [42]. Being unaware of signs or symptoms of cervical cancer may lead to a late diagnostic confirmation with an advanced stage of the disease [43]. Moreover, cultural norms, especially in Vietnam, may limit a woman’s ability to speak up or seek medical interventions if the symptoms are not clear [10].

Only half of the participants knew about the human papillomavirus (HPV) vaccine that can reduce the chances of developing cervical cancer. This percentage was lower than those from a study conducted among Vietnamese women, who believed that their knowledge about cervical cancer and HPV vaccines was sufficient to make a decision about vaccination [44]. Another study revealed that inadequate knowledge about the magnitude and causes of cervical cancer may contribute to incomplete vaccination, and a majority of respondents had not heard of the HPV vaccine prior to recommendations from a doctor [45]. As behavioral intentions are greatly impacted by the expected health outcomes and subjective assessment of the risks and advantages of those outcomes, providing information about favorable benefit-risk considerations in HPV vaccine recommendations are necessary to increase women’s ability to make an informed decision about vaccination [46]. Furthermore, it is important to look into social networks that influence one’s health knowledge that can lead to health decisions. In our study, having one sexual partner was positively associated with having greater knowledge of both breast cancer and cervical cancer compared to having no sexual partners or not being sexually active within the last 12 months. In terms of where participants received their health information, receiving information from friends or relatives was associated with having greater knowledge of cervical cancer.

Some limitations of this study should be acknowledged. First, self-reporting data may lead to recall bias as well as social desirability bias. Second, cross-sectional study design may limit the ability to identify causal relationships between associated factors and the knowledge of participants. Furthermore, although we had conducted this study in three different factories, the generalization of our results cannot be established due to convenience sampling methods.

Despite the limitations described, several implications can be drawn from the research. First, interventions for early detection and reduction of breast cancer and cervical cancer should be expanded to industrial workers, especially interventions that can increase knowledge about the causes and common signs or symptoms of these conditions among female workers. Such interventions may help prevent modifiable risk factors of breast and cervical cancers related to one’s lifestyle. Second, health communication campaigns should focus on people who have not had sexual intercourse or are not sexually active, since we found that these workers are at higher risk of having insufficient breast cancer and cervical cancer knowledge. Third, future education campaigns to ensure proper awareness about HPV vaccination and breast cancer prevention should be developed for factory workers. Finally, the internet should be explored as a medium to combat potentially inaccurate health information about breast and cervical cancer among workers.

## 5. Conclusions

Our study suggested a low to moderate level of knowledge about breast cancer and cervical cancer, especially regarding the causes and common signs or symptoms of these diseases, among industrial workers. One important finding from this study is also limited awareness of the HPV vaccine to prevent cervical cancer. Developing targeted education campaigns is needed to further ensure a proper understanding of the cancers and their prevention strategies among this vulnerable population.

## Figures and Tables

**Table 1:** Demographic and working characteristics of respondents.

Characteristics	*n*	%
Gender		
Male	48	16.7
Female	239	83.3
Education		
Under High School	25	8.7
High School	176	61.3
Above High School	86	30.0
Marital Status		
Single	12	4.2
Living with Spouse/Partner	277	95.9
Number of children		
0	12	4.4
1	44	16.0
2	177	64.1
>2	43	15.6
Nonimmigrant/Migrant	137	48.9
	Mean SD	
Age (Years)	31.9	4.5
Monthly Income (USD)	273.6	96.5
Years of Experience	9.9	3.8
Working Hours Per Day	8.2	0.9

**Table 2:** Health status and health risk behaviors.

Characteristics	Male		Female		Total		*p*-Value
	*n*	%	*n*	%	*n*	%	
Number of Sexual Partners within Last 12 Months							
0	5	11.6	33	17.7	38	16.6	0.57 ^†^
1	31	72.1	120	64.5	151	65.9	
Did not remember	7	16.3	33	17.7	40	17.5	
Current Smoking Status							
Never Smokers	20	42.6	204	95.8	224	86.2	<0.01 *
Former Smokers	8	17.0	9	4.2	17	6.5	
Current Smokers	19	40.4	0	0.0	19	7.3	
Current Drinking Status							
Hazardous Drinking	35	76.1	3	1.3	38	13.7	<0.01 *
Binge Drinking	34	73.9	3	1.3	37	13.4	<0.01 *
Other Medical Characteristics							
Having Acute or Chronic Conditions	45	95.7	187	82.4	232	84.7	0.02 *
Having Medical Checkup in the Last 12 Months	41	93.2	215	94.3	256	94.1	0.77 *
Using Reproductive Healthcare and Premarital Services in the Last 12 Months	14	31.1	111	48.9	125	46.0	0.03 *
Health Information Sources							
Friends/Relatives	18	40.0	104	44.3	122	43.6	0.6 *
Poster/Banner	6	13.3	15	6.4	21	7.5	0.11 *
Internet	25	55.6	113	48.1	138	49.3	0.36 *
Mobile phone messages	9	20.0	17	7.2	26	9.3	<0.01 *
Radio, TV	20	44.4	82	34.9	102	36.4	0.22 *
Speakers	9	20.0	29	12.3	38	13.6	0.17 *
Magazines, newspaper	19	42.2	87	37.0	106	37.9	0.5 *
Medical staffs	26	57.8	114	48.5	140	50.0	0.26 *
Social media	25	55.6	109	46.4	134	47.9	0.26 *
Other	1	2.2	1	0.4	2	0.7	0.19 *

* Chi-square test; ^†^ Fisher-exact test.

**Table 3:** Knowledge of breast cancer.

Characteristics	*n*	%
Causes of Breast Cancer		
No Breastfeeding	90	32.3
Genetic	70	25.1
Pollution and Chemicals	110	39.4
Smoking	81	29.0
Mammary Gland Engorgement	91	32.6
Signs of Breast Cancer		
Feeling Lumps in the Breast	213	76.3
Breast Enlarging or Breast Shape Changing	128	45.9
Swollen Lymph Nodes in Armpit	103	36.9
One of the Nipples is Dimpled or Rough	70	25.1
Breast Skin Thick, Wrinkled	54	19.4
Breast Skin Changing Color or Grainy Like Orange Peel	53	19.0
Pus Spills from the Nipple	98	35.1
Prevention Measures for Breast Cancer		
Early Diagnosis of Breast Cancer	226	81.3
Breastfeeding	125	45.0
Healthy Diet	114	41.0
Exercise Regularly	127	45.7
No Drinking Alcohol	88	31.7
No Smoking	99	35.6
	**Mean**	**SD**
**Total Score (Score Ranged from 0 to 18)**	6.9	5.1

Cronbach’s alpha = 0.92.

**Table 4:** Knowledge of cervical cancer.

Characteristics	*n*	%
Sexually Transmitted Diseases Believed to Cause Cervical Cancer		
Gonorrhea	120	44.0
Syphilis	122	44.7
Warts	114	41.8
Chlamydia infection	114	41.8
Assessed Factors as the Cause of Cervical Cancer		
Genital Infections	217	77.5
Having A Lot of Sexual Partners	140	50.0
Early Sexual Initiation	68	24.3
Too Many Children	36	12.9
Smoking	41	14.6
Assessed Symptoms as Signs of Cervical Cancer		
Vaginal Bleeding after Sex	152	54.5
Abnormal Bleeding between Menstrual Cycles	102	36.6
Vaginal Discharge	120	43.0
Pain when Having Sex	114	40.9
Pelvic Pain	72	25.8
Heavy Bleeding during Menstruation	47	16.9
Measures Believed to Prevent Cervical Cancer		
Periodic Gynecological Examination, Gynecological Treatment	218	77.6
Vaccination	131	46.6
Not Having A Lot of Sexual Partners	131	46.6
Not Early Sexual Initiation	83	29.5
Cervical Cancer Cannot Be Prevented	4	1.4
	**Mean**	**SD**
**Total Score (Score Ranged from 0 to 19)**	7.9	5.0

Cronbach’s alpha = 0.92.

**Table 5:** Factors related to knowledge of breast and cervical cancer.

Characteristics	Knowledge of Breast Cancer		Knowledge of Cervical Cancer	
	Coefficient	95% CI	Coefficient	95% CI
Gender (Female vs. Male)	1.20 **	0.73; 1.67	0.97 ***	0.57; 1.37
Education (vs. Under High School)				
High School	−0.26	−0.68; 0.15	−0.16	−0.60; 0.27
Above High School	−0.22	−0.65; 0.21	−0.04	−0.47; 0.40
Marital Status (vs. Single)				
Living with Spouse/Partner	−1.13	−0.64; 0.42	−0.14	−1.54; 0.29
Immigrant (No vs. Yes)	−0.19	−0.44; 0.06	−0.12	−0.33; 0.09
Age	0.05	−0.03; 0.04	−0.03*	−0.06; 0.00
Years of Experience	0.02	−0.01; 0.05	0.03**	0.00; 0.06
Number of Sexual Partners within Last 12 Months (1 vs. 0)	14.38 ***	12.42; 16.34	2.17 ***	1.71; 2.55
Current Smoking Status (vs. Never Smokers)				
Former Smokers	0.56 ***	0.13; 0.98	0.45 *	−0.00; 0.93
Current Smokers	0.53 *	−0.29; 1.36	0.33	−0.17; 1.22
Medical Checkup in the Last 12 Months (Yes vs. No)	1.70 **	0.20; 3.24	0.05	−0.76; 0.85
Using Reproductive Healthcare and Premarital Services in the Last 12 Months (Yes vs. No)	0.07	−0.16; 0.30	0.02	−0.18; 0.24
Health Information Sources (Yes vs. No)				
Friends/Relatives	0.07	−0.19; 0.33	0.30 **	0.06; 0.55
Posters/Banners	0.36 *	−0.04; 0.70	0.10	−0.30; 0.45
Internet	1.66 ***	1.09; 2.22	0.12	−0.03; 0.30
Mobile Phone Messages	−0.25	−2.59; 0.43	−0.10	−0.60; 0.47
Radio, TV	−0.32 *	−0.64; 0.00	−0.15	−0.43; 0.12
Speaker	0.39 **	0.02; 0.74	0.10	−0.30; 0.63
Magazines, Newspaper	0.33 **	0.01; 0.63	0.17	−0.13; 0.33
Medical Staffs	0.70	−1.23; 2.74	0.08	−0.11; 0.33
Social Media	0.85	−0.25; 1.98	−0.02	−0.18; 0.12

*** *p* < 0.01, ** *p* < 0.05, * *p* < 0.1.

## Supplementary Materials

The following are available online at https://www.mdpi.com/1660-4601/16/21/4301/s1, S1: survey of health status of workers of industrial zones.

## References

[1] World Health Organization (2018). Breast Cancer.

[2] Fitzmaurice C., Dicker D., Pain A., Hamavid H., Moradi-Lakeh M., MacIntyre M.F., Allen C., Hansen G., Woodbrook R., Global Burden of Disease Cancer Collaboration (2015). The Global Burden of Cancer 2013. JAMA Oncol..

[3] Wadler B.M., Judge C.M., Prout M., Allen J.D., Geller A.C. (2011). Improving Breast Cancer Control via the Use of Community Health Workers in South Africa: A Critical Review. J. Oncol..

[4] Coleman M.P., Quaresma M., Berrino F., Lutz J.M., De Angelis R., Capocaccia R., Baili P., Rachet B., Gatta G., Hakulinen T. (2008). Cancer survival in five continents: A worldwide population-based study (CONCORD). Lancet Oncol..

[5] Bray F., Ferlay J., Soerjomataram I., Siegel R.L., Torre L.A., Jemal A. (2018). Global cancer statistics 2018: GLOBOCAN estimates of incidence and mortality worldwide for 36 cancers in 185 countries. CA Cancer J. Clin..

[6] World Health Organization (2018). Cervical Cancer.

[7] Jenkins C., Minh L.N., Anh T.T., Ngan T.T., Tuan N.T., Giang K.B., Hoat L.N., Lohfeld L., Donnelly M., Van Minh H. (2018). Breast cancer services in Vietnam: A scoping review. Glob. Health Action.

[8] Global Health Data Exchange (2017). Global Burden of Diseases.

[9] Ferlay J., Soerjomataram I., Dikshit R., Eser S., Mathers C., Rebelo M., Parkin D.M., Forman D., Bray F. (2015). Cancer incidence and mortality worldwide: Sources, methods and major patterns in GLOBOCAN 2012. Int. J. Cancer.

[10] Thi Nguyen D.N., Simms K., Vu Nguyen H.Q., Van Tran T., Nguyen N.H., LaMontagne D.S., Castle P., Canfell K. (2019). The burden of cervical cancer in Vietnam: Synthesis of the evidence. Cancer Epidemiol..

[11] Jia Y., Li S., Yang R., Zhou H., Xiang Q., Hu T., Zhang Q., Chen Z., Ma D., Feng L. (2013). Knowledge about cervical cancer and barriers of screening program among women in Wufeng County, a high-incidence region of cervical cancer in China. PLoS ONE.

[12] Shrestha S., Dhakal P. (2017). Knowledge, Attitude and Practice Regarding Cervical Cancer Screening Among Women Attending a Teaching Hospital, Bharatpur, Chitwan. J. Fam. Reprod. Health.

[13] Liu L.Y., Wang Y.J., Wang F., Yu L.X., Xiang Y.J., Zhou F., Li L., Zhang Q., Fu Q.Y., Ma Z.B. (2018). Factors associated with insufficient awareness of breast cancer among women in Northern and Eastern China: A case-control study. BMJ Open.

[14] Thapa N., Maharjan M., Petrini M.A., Shah R., Shah S., Maharjan N., Shrestha N., Cai H. (2018). Knowledge, attitude, practice and barriers of cervical cancer screening among women living in mid-western rural, Nepal. J. Gynecol. Oncol..

[15] The World Bank (2018). The World Bank in Vietnam.

[16] International Labour Organization (2018). Viet Nam: Serving Markets and Workers.

[17] Chee H.L., Rashidah S., Shamsuddin K., Zainiyah S.Y. (2003). Knowledge and practice of breast self examination and Pap smear screening among a group of electronics women workers. Med. J. Malays..

[18] Sung T.I., Chen P.C., Jyuhn-Hsiarn Lee L., Lin Y.P., Hsieh G.Y., Wang J.D. (2007). Increased standardized incidence ratio of breast cancer in female electronics workers. BMC Public Health.

[19] Savitz D.A., Andrews K.W., Brinton L.A. (1995). Occupation and cervical cancer. J. Occup. Environ. Med..

[20] Alterman T., Burnett C., Peipins L., Lalich N., Halperin W. (1997). Occupation and cervical cancer: An opportunity for prevention. J. Womens Health.

[21] Bradley K.A., DeBenedetti A.F., Volk R.J., Williams E.C., Frank D., Kivlahan D.R. (2007). AUDIT-C as a brief screen for alcohol misuse in primary care. Alcohol. Clin. Exp. Res..

[22] Ryan P. (2009). Integrated theory of health behavior change: Background and intervention development. Clin. Nurse Spec..

[23] Lorig K., Ritter P.L., Plant K. (2005). A disease-specific self-help program compared with a generalized chronic disease self-help program for arthritis patients. Arthritis Care Research..

[24] Gage H., Hampson S., Skinner T.C., Hart J., Storey L., Foxcroft D., Kimber A., Cradock S., McEvilly E.A. (2004). Educational and psychosocial programmes for adolescents with diabetes: Approaches, outcomes and cost-effectiveness. Patient Educ. Couns..

[25] Parsa P., Kandiah M., Mohd Zulkefli N.A., Rahman H.A. (2008). Knowledge and behavior regarding breast cancer screening among female teachers in Selangor, Malaysia. Asian Pac. J. Cancer Prev..

[26] Ames B.N., Gold L.S. (1998). The causes and prevention of cancer: The role of environment. Biotherapy.

[27] Brody J.G., Rudel R.A. (2003). Environmental pollutants and breast cancer. Environ. Health Perspect..

[28] Gupta A., Shridhar K., Dhillon P.K. (2015). A review of breast cancer awareness among women in India: Cancer literate or awareness deficit?. Eur. J. Cancer.

[29] Forbes L.J., Atkins L., Thurnham A., Layburn J., Haste F., Ramirez A.J. (2011). Breast cancer awareness and barriers to symptomatic presentation among women from different ethnic groups in East London. Br. J. Cancer.

[30] Aydogan U., Doganer Y.C., Kilbas Z., Rohrer J.E., Sari O., Usterme N., Yuksel S., Akbulut H., Balkan S.M., Saglam K. (2015). Predictors of knowledge level and awareness towards breast cancer among Turkish females. Asian Pac. J. Cancer Prev..

[31] Kobeissi L., Samari G., Telesca D., Esfandiari M., Galal O. (2014). The impact of breast cancer knowledge and attitudes on screening and early detection among an immigrant Iranian population in southern California. J. Relig. Health.

[32] Jenkins C., Ngan T.T., Ngoc N.B., Phuong T.B., Lohfeld L., Donnelly M., Van Minh H., Murray L. (2019). Strengthening breast cancer services in Vietnam: A mixed-methods study. Glob. Health Res. Policy.

[33] Bruce J.G., Tucholka J.L., Steffens N.M., Neuman H.B. (2015). Quality of online information to support patient decision-making in breast cancer surgery. J. Surg. Oncol..

[34] Trieu P.D., Mello-Thoms C., Brennan P.C. (2015). Female breast cancer in Vietnam: A comparison across Asian specific regions. Cancer Biol. Med..

[35] Demment M.M., Peters K., Dykens J.A., Dozier A., Nawaz H., McIntosh S., Smith J.S., Sy A., Irwin T., Fogg T.T. (2015). Developing the Evidence Base to Inform Best Practice: A Scoping Study of Breast and Cervical Cancer Reviews in Low- and Middle-Income Countries. PLoS ONE.

[36] Jenkins C.N., McPhee S.J., Bird J.A., Pham G.Q., Nguyen B.H., Nguyen T., Lai K.Q., Wong C., Davis T.B. (1999). Effect of a media-led education campaign on breast and cervical cancer screening among Vietnamese-American women. Prev. Med..

[37] Im E.O., Park Y.S., Lee E.O., Yun S.N. (2004). Korean women’s attitudes toward breast cancer screening tests. Int. J. Nurs. Stud..

[38] Sambanje M.N., Mafuvadze B. (2012). Breast cancer knowledge and awareness among university students in Angola. Pan Afr. Med. J..

[39] Aweke Y.H., Ayanto S.Y., Ersado T.L. (2017). Knowledge, attitude and practice for cervical cancer prevention and control among women of childbearing age in Hossana Town, Hadiya zone, Southern Ethiopia: Community-based cross-sectional study. PLoS ONE.

[40] Mitiku I., Tefera F. (2016). Knowledge about Cervical Cancer and Associated Factors among 15–49 Year Old Women in Dessie Town, Northeast Ethiopia. PLoS ONE.

[41] Ali-Risasi C., Mulumba P., Verdonck K., Vanden Broeck D., Praet M. (2014). Knowledge, attitude and practice about cancer of the uterine cervix among women living in Kinshasa, the Democratic Republic of Congo. BMC Womens Health.

[42] Touch S., Oh J.K. (2018). Knowledge, attitudes, and practices toward cervical cancer prevention among women in Kampong Speu Province, Cambodia. BMC Cancer.

[43] Taylor V.M., Nguyen T.T., Jackson J.C., McPhee S.J. (2008). Cervical cancer control research in Vietnamese American communities. Cancer Epidemiol. Biomark. Prev..

[44] Paul P., LaMontagne D.S., Le N.T. (2012). Knowledge of cervical cancer and HPV vaccine post- vaccination among mothers and daughters in Vietnam. Asian Pac. J. Cancer Prev..

[45] Yi J.K., Lackey S.C., Zahn M.P., Castaneda J., Hwang J.P. (2013). Human papillomavirus knowledge and awareness among Vietnamese mothers. J. Community Health.

[46] Darabi F., Yaseri M., Kaveh M.H., Khalajabadi Farahani F., Majlessi F., Shojaeizadeh D. (2017). The effect of a theory of planned behavior-based educational intervention on sexual and reproductive health in iranian adolescent girls: A randomized controlled trial. J. Res. Health Sci..

