# Supplementary material for: Knowledge towards Cervical and Breast Cancers among Industrial Workers: Results from a Multisite Study in Northern Vietnam
Source: Int J Environ Res Public Health. 2019 Nov 5;16(21):4301. doi: 10.3390/ijerph16214301 (PMC6862634; doi:10.3390/ijerph16214301)
Supplement: Supplementary file 1 [file ijerph-16-04301-s001.pdf]

SURVEY OF HEALTH STATUS OF WORKERS OF INDUSTRIAL ZONES

A	GENERAL INFORMATION	
A1	**The name of the company you are currently working at**	…………………..
A3	**Year of birth?**	…………………
A4	**What is your gender?**<br>Male<br>Female	 1<br>2
A5	**What is your highest educational level?**<br>Do not go to school<br>Primary school<br>Junior high school<br>High school<br>Intermediate, college, vocational training<br>University<br>Post-graduated	 1<br>2<br>3<br>4<br>5<br>6<br>7
A6	**What is your marital status?**<br>Single<br>Live with spouse<br>Living as a spouse, unmarried<br>Divorced / Separated<br>Widow	 1<br>2<br>3<br>4<br>5
A7	**How many children do you have?**	……… children
A8	**Are you a immigrants?** **Local people**<br>**Immigrant**	1<br>2
A9	**For the past 1 year, your average monthly personal income**	…………...VND
**B**	**WORKING STATUS**	
B1	**How long have you worked in here**	…………. Year<br>………….. month
B2	**How many working hours per day?**	……………. Hour

C	HEALTH STATUS	
C1	**DO you have any health problems (illnesses or symptoms)? (Choose multiple answers)**<br>High blood pressure<br>Heart<br>Diabetes<br>Asthma<br>Stomach / digestive<br>Chronic obstructive pulmonary<br>Allergy<br>Dermatology<br>Ear / hearing diseases<br>Lumbar spine pain / Cervical spine pain<br>Osteoarthritis pain<br>Headache<br>Shortness of breath<br>Other:……………………………………<br>There is no disease / disability	 1<br>2<br>3<br>4<br>5<br>6<br>7<br>8<br>9<br>10<br>11<br>12<br>13<br>14<br>99
C2	**Recently, how often do you drink alcohol?**<br>Never<br>Monthly<br>Weekly<br>2-3 times per week<br>> = 4 times per week	 1<br>2<br>3<br>4<br>5
C3	**Normally, how many cups / glass do you usually drink each time?**	………..cup/glass
C4	**How often do you drink 6 or more cups of alcohol?**<br>**Never**<br>**Every few months**	 1<br>2

	**Monthly**<br>**Weekly**<br>**Daily**	3<br>4<br>5
**C5**	**Have you ever smoked a total of 100 cigarettes or more? (100 cigarettes = 5 packs of cigarettes) ?**<br>**Yes**<br>**Is not**<br>**Do not know**	 <br>1<br>2<br>99
**C6**	**In the past 30 days, did you smoke?**<br>**Yes**<br>**No**	 1<br>2
**C7**	**In the last 12 months, how many partners did you have sex with (your spouse / lover / partner) paid / your partner received money?**<br>**Do not remmenber**<br>**Do not have sex**	 …………people<br>98<br>99

**D**	**HEALTH SERVICE UTILIZATION**	
**D1**	**In the past 12 months, did you or do you have regular checkups?**<br>Yes<br>No	 1<br>2
**D2**	**In the past 12 months, have you ever used counseling and reproductive health care services?**<br>Yes<br>No	 1<br>2
**D3**	**How do you usually access health information?** (Choose multiple answers)<br>Friends / relatives<br>Posters / banner<br>Internet<br>Text message<br>Radio and television<br>Loudspeaker<br>Newspapers, books<br>Medical staff<br>Social Network<br>Other (specify) ……………………………………….. ..	 1<br>2<br>3<br>4<br>5<br>6<br>7<br>8<br>9<br>10

**E**	**KNOWLEDGE**	
**E1**	**What causes breast cancer? (Choose multiple answers)**<br>Do not breastfeed<br>Genetic<br>Pollution of dust and chemicals<br>Smoke<br>Blocked breast milk<br>Unknown / Unknown	 1<br>2<br>3<br>4<br>5<br>6
**E2**	**What are the warning signs of breast cancer? (Choose multiple answers)**<br>Feeling a lump in the breast<br>Breast enlargement or changes in breast shape<br>Swollen lymph nodes<br>One of the nipples is dimpled or shaggy<br>Skin of the breast is thicker, wrinkled<br>or change color, grainy like orange peel<br>Pus oozing from the nipple<br>Unknown / Unknown	 1<br>2<br>3<br>4<br>5<br>6<br>7<br>8
**E3**	**What are the breast cancer prevention measures? (Choose multiple answers)**<br>Screening and diagnosis of breast cancer early and periodically<br>Grow kids by mom milk<br>Diet<br>Exercise regularly<br>Do not drink alcohol<br>No smoking<br>Unknown / Unknown	 1<br>2<br>3<br>4<br>5<br>6<br>7
**E4**	**What causes cervical cancer? (Choose multiple answers)**	

	Inflammation of the genital tract<br>Having sex with many people<br>Having sex early<br>Spawning a lot<br>Smoke<br>Unknown / Unknown	1<br>2<br>3<br>4<br>5<br>6
**E5**	**Signs of cervical cancer? (Choose multiple answers)**<br>Vaginal bleeding after sex<br>Any bleeding between menstrual periods<br>Vaginal discharge<br>Pain during sex<br>Pelvic pain,<br>Heavy bleeding during menstruation<br>Unknown / Unknown	 1<br>2<br>3<br>4<br>5<br>6<br>7
**E6**	**Measures to prevent cervical cancer? (Choose multiple answers)**<br>Periodic gynecological examination and treatment of gynecological diseases (if any)<br>Vaccination<br>Do not have sex with many people<br>Do not have sex early<br>Other:…………………………..<br>Not preventable<br>Unknown / Unknown	 1<br>2<br>3<br>4<br>5<br>6<br>7

PLEASE THANK YOU FOR YOUR PARTICIPATION!
